# Swainsonine reduces 5-fluorouracil tolerance in the multistage resistance of colorectal cancer cell lines

**DOI:** 10.1186/1476-4598-6-58

**Published:** 2007-09-21

**Authors:** Jun Hamaguchi, Hiroaki Nakagawa, Masato Takahashi, Takeaki Kudo, Naoya Kamiyama, Bailong Sun, Takahiro Oshima, Yuji Sato, Kisaburo Deguchi, Satoru Todo, Shin-Ichiro Nishimura

**Affiliations:** 1 Department of General Surgery, Graduate School of Medicine, Hokkaido University, Sapporo 060-8638, Japan; 2 Graduate School of Advanced Life Science, Hokkaido University, Sapporo 001-0021, 001-0021, Japan; 3 Department of Sensory Physiology, Asahikawa Medical College, Asahikawa 078-8510, Japan

## Abstract

**Background:**

Drug resistance is a major problem in cancer chemotherapy. Acquisition of chemo-resistance not only reduces the effectiveness of drugs, but also promotes side effects and markedly reduces the patient's quality of life. However, a number of resistance mechanisms have been reported and are thought to be the reason for the difficulties in solving drug-resistance problems.

**Result:**

To investigate the mechanisms of drug resistance, a set of cell lines with different levels of sensitivity and possessing different mechanisms of resistance to 5-fluorouracil (5-FU) was established from a colorectal cancer cell line. The expression of thymidylate synthase, orotic acid phosphoribosyltransferase and dihydropyrimidine dehydrogenase, which are well known to be related to drug resistance, differed among these cell lines, indicating that these cell lines acquired different resistance mechanisms. However, swainsonine, an inhibitor of N-glycan biosynthesis, reduced 5-FU-tolerance in all resistant cells, whereas the sensitivity of the parental cells was unchanged. Further analysis of the N-glycan profiles of all cell lines showed partial inhibition of biosynthesis and no cytotoxicity at the swainsonine dosage tested.

**Conclusion:**

These observations suggest that N-linked oligosaccharides affect 5-FU resistance more widely than do drug-resistance related enzymes in colorectal cancer cells, and that the N-glycan could be a universal target for chemotherapy. Further, swainsonine may enhance the performance of chemotherapy by reducing tolerance.

## Background

Colorectal cancer has one of the highest incidences among all forms of cancer around the world. In many cases, the cancer cannot be completely controlled by surgical intervention, so multidisciplinary treatment including chemotherapy is required. However, cancer cells often acquire drug resistance during treatment and a patient's prognosis can become very unfavorable [1]. Acquisition of chemo-resistance not only reduces the effectiveness of drugs, but also promotes side effects and markedly reduces the patient's quality of life.

5-fluorouracil (5-FU), a fluorinated pyrimidine, is a key anti-colorectal cancer drug. 5-FU affects the synthesis and repair of DNA and RNA processing in cancer cells [2-4]. 5-FU metabolic enzymes, such as thymidylate synthase (TS), orotic acid phosphoribosyltransferase (OPRT), uridine phosphorylase (UP), dihydropyrimidine dehydrogenase (DPD), and pyrimidine nucleoside phosphorylase (PyNPase), are thought to play a role in the resistance mechanism [5-8].

Oligosaccharides on glycoproteins mediate a dynamic protein state, involving folding, quality control, secretion and catabolism [9,10]. Glycans are also related to tumor progression and metastasis as well as to immune system activity [11], and their potential relationship to chemo-resistance has recently been examined [12,13]. In N-glycan biosynthesis, a precursor high-mannose type oligosaccharide, consisting of 2 N-acetylglucosamine (GlcNAc), 9 mannose (Man) and 3 glucose (Glc) residues, is synthesized on a dolichol di-phosphate and transferred to a nascent polypeptide in the endoplasmic reticulum. During protein folding, one Man and three Glc residues are removed to form an M8.1 high-mannose type N-glycan. This oligosaccharide residue functions as a tag to carry correctly folded glycoproteins to the Golgi apparatus, while misfolded proteins are recognized by the protein degradation system [14]. In the Golgi, α-mannosidase I removes a further 3 Man residues from M8.1 to form M5.1, then N-acetylglucosaminyltransferase I attaches a GlcNAc residue to M5.1, forming the hybrid-type oligosaccharide. Next, α-mannosidase II removes two Man residues and N-acetylglucosaminyltransferase II adds another GlcNAc to form complex-type N-glycans. These glycans are modified by galactose, fucose and sialic acid residues to form a variety of oligosaccharide structures [15]. Swainsonine, a known glycosylation inhibitor [16,17], inhibits α-mannosidase II activity in the N-glycan biosynthesis pathway and blocks production of complex-type oligosaccharides [18,19]. Swainsonine has been of great use in the study of N-glycan functions, with many important results published since its discovery [20-22]. The anti-tumor activity of swainsonine has also been previously examined [23]. Swainsonine exhibits not only cytotoxicity, but inhibits cancer cell metastasis [24,25], decreases the toxicity of chemotherapeutic drugs [26,27] and works as immunomodulator [28,29]. Despite its side effects, clinical studies on patients have shown that swainsonine is of some benefit as a chemotherapeutic drug [30,31], suggesting that it might have further applications in this field. Tunicamycin, which inhibits N-glycosylation, has been shown to enhance sensitivity to cisplatin [32] and reduce drug-resistance in multidrug-resistant carcinoma cells [33].

We established various gradations of 5-FU resistant cell lines from a mouse colon cancer cell line and analyzed the expression enzymes related to resistance, the effect of swainsonine and the glycoforms present in those cells.

## Results

### Establishment of 5-FU resistant murine colorectal cell lines

We established various gradations of 5-FU resistant cell lines from the mouse colon cancer cell line, colon 26. The morphology of the 5-FU resistant cells was similar to that of the parental line, and their proliferation rates were similar except for L0-500, which proliferated slightly more rapidly and aggregated more easily, resulting in cell death (data not shown).

**Figure 1:**
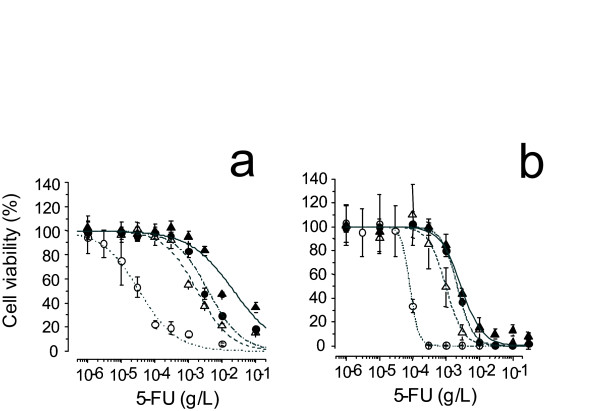
**Growth inhibition curves of L0 (colon26), L0-200, L0-500 and L0-1000 treated with 5-FU**. **a**, Cytotoxic effect of 5-FU was estimated by MTT assay. **b**, Cell growth inhibitory effects of 5-FU were estimated by CD-DST. Means of L0, L0-200, L0-500 and L0-1000 are represented by open circles, open triangles, closed circles and closed triangles, respectively, the range of S.D. from 6 measurements is indicated by vertical bars

Drug resistance in the parental L0 line and in the L0-200, L0-500 and L0-1000 lines was assayed using cytotoxicity assay and growth inhibition assay. Fifty percent inhibition concentration (IC-50) values of the L0, L0-200, L0-500 and L0-1000 lines against 5-FU were 0.110, 1.07, 3.87 and 4.86 μg/mL, respectively, by Mossman's 3-(4,5-dimethyl-2-thiazolyl)-2,5-diphenyl-2H-tetrazolium bromide (MTT) assay (Fig. 1a). To estimate the effect of growth inhibition, we employed an alternative cytotoxicity assay, the collagen gel droplet-embedded culture drug sensitivity test (CD-DST) assay. IC-50 values of the L0, L0-200, L0-500 and L0-1000 lines using this assay were 0.0810, 0.970, 2.20 and 2.30 μg/mL (Fig. 1b), respectively. After maintenance for 6 months in the absence of 5-FU, the cell lines exhibited only slightly reduced 5-FU resistance (data not shown).

### Expression of proteins related to 5-FU resistance

**Figure 2:**
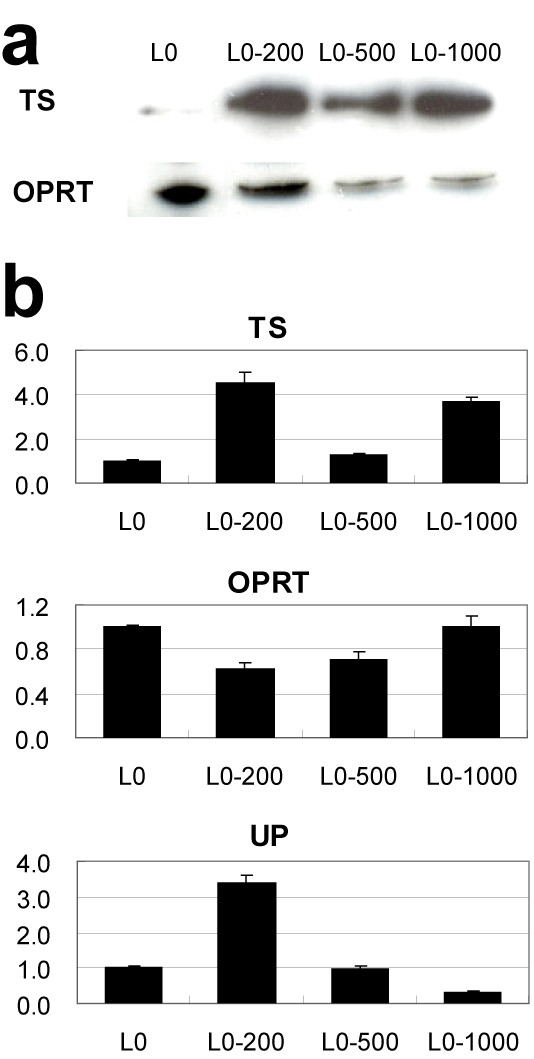
**Changes in 5-FU-related enzymes in resistant sublines**. **a**, Western blotting analyses of TS and OPRT proteins. **b**, Real-time PCR analyses of TS, OPRT, TS and UP proteins. mRNA expression of each enzyme is shown as the ratio relative to the parental L0 (colon 26) cell. Each column represents the mean with S.D. of 4 measurements.

TS is reported to be a 5-FU catabolic enzyme, and OPRT catalyzes 5-FU to 5-fluorouracil mono-phosphate, which is finally taken into RNA where it inhibits RNA functions. Thus, we used Western-blotting to measure OPRT and TS protein levels in the resistant lines. OPRT was down-regulated and TS was up-regulated in all 5-FU resistant sublines; L0-200, L0-500 and L0-1000 (Fig. 2a).

As TS, OPRT, and UP expression are correlated with 5-FU resistance, we used real-time RT-PCR to analyze the expression of these factors in the resistant lines. TS expression was up-regulated in the 5-FU resistant cell lines, though the change was only slight in the L0-500 line. OPRT expression was slightly down-regulated in the 5-FU resistant cell lines. UP expression in the L0-200 line was up-regulated 3-fold over that in the parental line; however, its expression in the L0-500 line was the same as that in the parental line, and it was down-regulated in the L0-1000 line, indicating that there was no overall linear relationship between 5-FU resistance and OPRT, TS, or UP expression (Fig. 2b). Expression of DPD, TP, and PyNPase could not be measured, probably due to the low levels of expression.

### Effect of a swainsonine on 5-FU resistant cells

**Figure 3:**
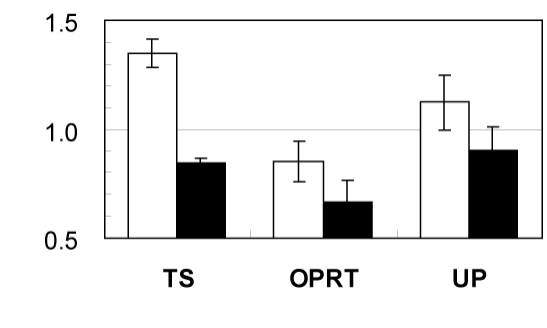
**Effects of swainsonine on sensitivity to 5-FU and mRNA expression of 5-FU-related enzymes**. mRNA expression of TS, OPRT and UP following swainsonine treatment of L0 (open column) and L0-1000 (closed column) measured by real-time RT-PCR. Data represent the ratio relative to the parental L0 cell without swainsonine. Each column represents the mean with S.D. of 4 measurements.

Swainsonine blocks α-mannosidase II activity, which is necessary to convert N-glycans from hybrid-type to complex-type. 5-FU resistant L0-1000 and parent cells show the same sensitivity to swainsonine. Over 90% of cells from both cell lines were killed by treatment with 30 μg/mL of swainsonine, whereas 80% of cells survived at 10 μg/mL swainsonine. Therefore, we applied 5 μg/mL swainsonine, at which concentration no cytotoxicity was observed. Swainsonine treatment significantly reduced the IC-50 against 5-FU of the resistant cells, but not that of the parental L0 line (Table 1). Real-time reverse transcription polymerase chain reaction (RT-PCR) showed that expression of TS was increased in the parental cell line and decreased in the resistant L0-1000 line by swainsonine treatment, whereas OPRT expression was decreased in both lines and UP expression was not affected (Fig. 3).

**Table 1:** Effects of swainsonine on the sensitivity to 5-FU in the parental and resistant sublines.

	Swainsonine (-)		Swainsonine (+)	
	
	IC-50 (μg/mL)	Ratio	IC-50 (μg/mL)	Ratio
L0 (colon 26)	0.110	1.00	0.110	1.00
L0-200	1.07	9.70	0.650	5.91
L0-500	3.87	35.1	1.45	13.2
L0-1000	4.86	44.2	3.01	27.4

IC-50s were calculated from the cell viability data obtained by MTT assay (n = 6).

### N-glycan profiling

**Figure 4:**
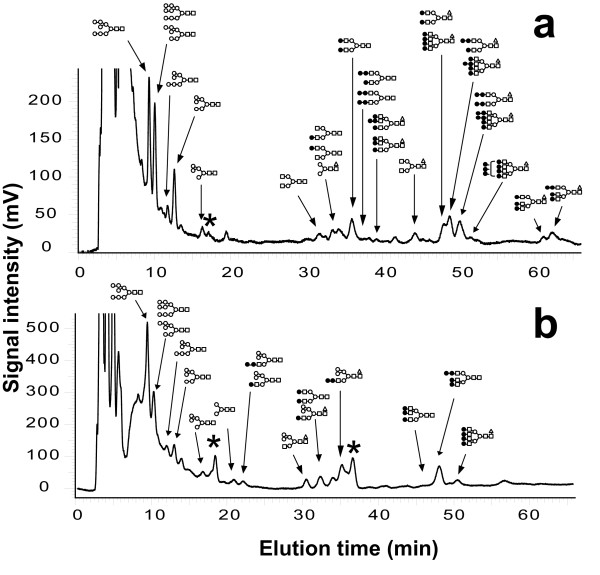
**Glycoform analyses by HPLC**. ODS chart pattern of L0 treated without (**a**) and with (**b**) swainsonine. Each peak corresponds to N-glycans illustrated in the chart. Closed square, GlcNAc; open circle, Man; closed circle, Gal; open triangle, Fuc.

**Figure 5:**
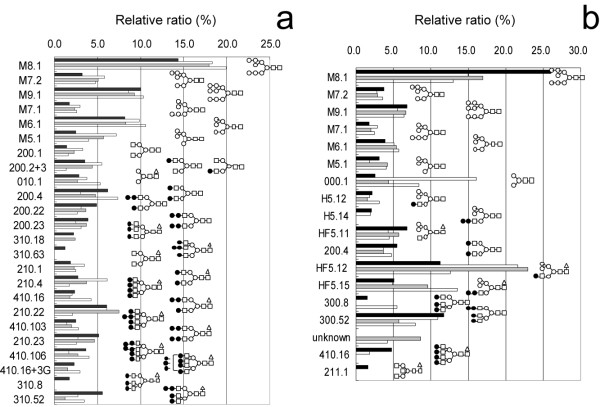
**N-glycan profiles from parental and resistant sublines treated without (a) and with (b) swainsonine**. L0 (closed column), L0-200 (open column), L0-500 (shaded column) and L0-1000 (striped column). Code numbers of oligosaccharide structures described in the reference [34].

**Figure 6:**
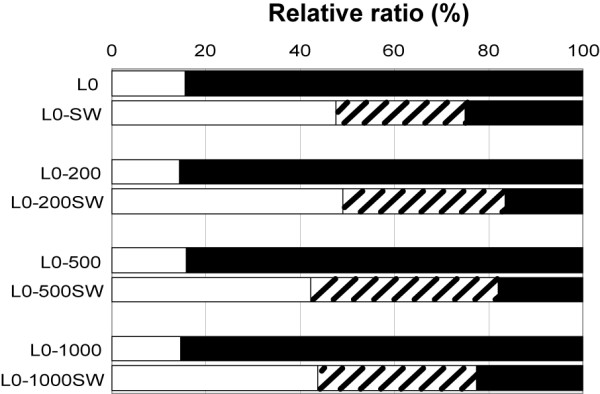
**Composition of N-glycans in parental and resistant sublines treated with or without swainsonine**. Swainsonine-treated L0, L0-200, L0-500 and L0-1000 are shown as L0 SW, L0-200SW, L0-500SW and L0-1000SW, respectively. Forms of N-glycans; high mannose (open column), hybrid (striped column) and complex (closed column).

N-glycans in the resistant lines were analyzed using a 2-dimensional mapping method combined with exoglycosidase digestion. Oligosaccharides were first separated on an octadecylsilyl (ODS) column using high performance liquid chromatography (HPLC) (Fig. 4), then each peak separated on the ODS was further analyzed on an amide column. Oligosaccharides were assigned their structure through a comparison of their elution positions with those previously referenced [34] (Fig. 5). N-glycans are classified into three types; high-mannose, hybrid and complex, according to their terminal monosaccharides. The ratios of glycan types were calculated and are shown in Figure 6.

## Discussion

Herein we describe a set of multistage 5-FU resistant cell lines: L0-200, L0-500 and L0-1000. These lines are useful in examining the mechanisms of drug resistance, as they are of the same genetic background and utilize different acquired mechanisms of drug resistance. The IC-50 values of the L0-200, L0-500 and L0-1000 lines against 5-FU were 9.7, 35 and 44 times, respectively, greater than that of the parental L0 line by MTT assay, and the 5-FU concentrations in the respective cell lines were 5.4, 7.7 and 4.9 times greater than that in the maintenance media.

To analyze the mechanisms underlying 5-FU resistance, we assayed the expression of 5-FU catabolic enzymes by Western blotting and real-time RT-PCR. Western blotting showed that TS expression was up-regulated whereas that of OPRT was down-regulated in the resistant cells compared to that in the parental line. This expression profile corresponds to that in previous reports showing that TS inactivates 5-FU, and OPRT activates 5-FU function [35-37]. OPRT expression was correlated with the degree of drug resistance, but TS expression in the L0-200 cell line was stronger than that in the L0-500 or L0-1000 lines. However, the results of real-time RT-PCR analysis did not correspond with those of Western blotting. The expression of TS mRNA in the L0-500 cells was almost same as that in the L0 parent cells. L0-1000 cells expressed little OPRT protein, but OPRT mRNA was expressed at the same level as in the L0 cells. These discrepancies might be due to the time-lag between protein and mRNA expression and the rate of protein degradation. Nonetheless, UP transcripts were most significantly up-regulated in the L0-200 cells, but were unchanged from the parental line in the L0-500 cells, and were down-regulated in the L0-1000 cells. Such differential expression indicates that the respective lines utilize different mechanisms of 5-FU resistance, including mechanisms not analyzed in this study; e.g., the degradation pathways of these enzymes. Such variations in the resistance mechanisms were expected and are thought to be the reason for the difficulties in solving drug-resistant mechanisms experienced to date.

In our study, a low-dosage swainsonine treatment, which shows no cytotoxicity, increased 5-FU sensitivity and altered glycoforms in the resistant cell lines. In contrast, the 5-FU sensitivity of the parental line was unchanged following swainsonine treatment. N-glycan profiles of both the resistant and parent cells were changed by swainsonine treatment. This result shows that swainsonine rescues chemotherapeutic drugs from a range of resistance mechanisms. In all cell lines, the proportion of complex-type N-glycans was decreased from 80% to 20% (Fig. 6), though the continued presence of complex-type N-glycans indicates that swainsonine did not block biosynthesis completely. Mice are known to perish without α-mannosidase II activity [38,39], but are able to survive with about 20% complex-type oligosaccharides [39,40]. Therefore, monitoring of the N-glycan profile would be an important step in the prevention of side-effects and would increase our understanding of resistance mechanisms. In this study, we analyzed N-glycan structures in detail in an attempt to determine diagnostic oligosaccharide structures related to drug-resistance. We could not confirm any common resistance-related structural characteristics in the oligosaccharides, but clearly observed that the mechanisms of 5-FU resistance were affected by alterations in the N-glycan structure. Recently, it has been reported that tunicamycin enhances the therapeutic effect of drugs in multi-resistant cancer cells [33]. Tunicamycin inhibits N-glycosylation and affects the protein folding process in the endoplasmic reticulum, whereas swainsonine takes effect after the completion of this process. A comparison of the mechanisms by which these two inhibitors enhance the effects of chemotherapeutic drugs is necessary in the future; however, these results confirm the potential importance of both. It has been shown that tunicamycin enhances the effect of several drugs in multi-drug resistant cells. Our study indicated that swainsonine may be universally effective against a range of different mechanisms for acquired drug-resistance, thereby simplifying the design of chemotherapy for individual patients. No lethal side effects have been observed in clinical trials using swainsonine, but the side-effects have outweighed the clinical advantages to date [30,31]. Our results, however, suggest that the administration of swainsonine can prevent acquired drug-resistance through the maintaining the effectiveness of chemotherapeutic reagents, and a reduction in the swainsonine concentration can reduce the side-effects caused by the treatment. Clinical studies should be undertaken to further examine this proposed mechanism. Studies on reducing drug-resistance have examined MS209, a P-glycoprotein inhibitor, but found that it was unsatisfactory in combating drug resistance [41,42]. Swainsonine, on the other hand, can be effective against various mechanisms of resistance and its side effects, pharmacokinetics and biochemical responses have been clarified in previous clinical trials [30,31]. Further, on the basis of previous studies, swainsonine is expected to control metastasis [24,25], decrease toxicity of drugs [26,27] and enhance immune systems [28,29]. Thus, the role of swainsonine as well as other glycosylation inhibitors in cancer therapy should be reviewed.

## Conclusion

Various gradations of 5-FU resistant cell lines acquired different mechanisms of 5-FU resistance; however, swainsonine was universally effective in increasing the sensitivity to 5-FU of all resistant cells. This result indicates that N-glycan biosynthesis is a better target for the prevention of 5-FU resistance, and points the way to the development of new chemotherapeutic strategies. To date, swainsonine has not been used as an anti-cancer drug, but this study presents a new strategy by which a low dosage of swainsonine can be used to recover the effectiveness of 5-FU or other chemotherapeutic reagents in tumors with acquired resistance.

## Methods

### Establishment of 5-FU resistant cell lines

Colon 26, a murine colorectal cancer cell line [43], was very kindly provided by Dr. Tatsuji Kataoka of The Japanese Foundation for Cancer Research. Colon 26 cells were maintained in RPMI-1640 medium (Gibco BRL, Rockville, MD) supplemented with 10% heat-inactivated fetal bovine serum (Gibco), 60 U/mL penicillin, and 30 mg/L streptomycin (Gibco), in a humidified 5% CO_2 _incubator at 37°C. To establish resistant lines, 5-FU (Kyowa Hakko Kogyo Co., Ltd., Tokyo, Japan) was added to the culture medium at an initial concentration of 25 ng/mL. Surviving cells were harvested and seeded onto another dish with 5-FU-containing media [44]. The concentration of 5-FU was increased every few weeks. The parental line was named "L0", and the 5-FU resistant lines were identified on the basis of the 5-FU concentration (ng/mL): for example, a line maintained in 200 ng/mL 5-FU was designated L0-200. Four lines, L0, L0-200, L0-500 and L0-1000, were established and the results are reported herein.

### Cytotoxicity (cell death) assay

Drug resistance was measured using MTT assay [45]. All cells were grown in RPMI-1640 medium supplemented with 20% heat-inactivated fetal bovine serum, 60 U/mL penicillin, and 30 mg/L streptomycin (Gibco), in a humidified 5% CO_2 _incubator at 37°C. A 50 μL suspension of 1.0 × 10^3 ^cells in culture medium was plated onto each well of a 96-well plate, and pre-incubated for 24 h. Then, 50 μL of medium including 5-FU was added and incubated for 70 h. Subsequently, 50 μg MTT (DOJINDO, Kumamoto, Japan) in PBS was added to a final concentration of 5 μg/mL and incubated for 2 h. After removal of the medium, cell membranes were permeabilized by the addition of 100 μL of dimethyl sulfoxide per well, and MTT formazan released from cells was measured by absorbance at 490 and 650 nm. Cytotoxicity was evaluated from the IC-50 value calculated using Origin 6.1J software (LightStone Co. Ltd., Tokyo, Japan).

### Growth inhibition assay

To verify drug resistance, we employed an alternative CD-DST method [46]. A collagen solution was made using a collagen gel culture kit (Nitta Gelatin Inc., Osaka, Japan) and 30 μL of cell suspension at 2 × 10^4 ^cells/mL was added to 30 μL of the collagen solution, drops were hung in 6-well culture plates, and the plates were then placed in a 5% CO_2 _incubator at 37°C for 1 h to form a gel. Three ml of culture medium was added to each well. After preincubation for 24 h, 5-FU was added. After an additional 24 h, each well was washed twice with 4 mL of PBS by gentle shaking in the incubator for 10 min. After removal of the PBS, 4 mL of culture medium was added to each well, and cells were cultured for 6 days. Neutral red was then added to each well at 50 μg/mL. Cells were fixed 45 min later with 10% neutral-buffered formalin and washed with water for 10 minutes. Finally, collagen gel droplets were air-dried and quantified by Primage^® ^(Nitta Gelatin) using an imaging apparatus.

### Swainsonine treatment

Swainsonine (Sigma) was added at 5 μg/mL to the medium when cells were harvested, and cells were incubated with the inhibitor for 5 days prior to the MTT assays, real-time RT-PCR, and glycoform analysis. Real-time RT-PCR was undertaken with the L0 and L0-1000, and the data were analyzed as the ratio of transcript levels derived from swainsonine-treated cells to those from untreated cells.

### Western blotting

Antibodies against OPRT and TS were provided by Taiho Pharmaceutical Co., Ltd (Tokyo, Japan). Protein was extracted from 1 × 10^6 ^cells of each line using CelLytic/Extraction Reagent (SIGMA), and 1.5 μg of protein, measured using the Bio-Rad DC protein assay kit (Hercules, CA), was loaded on a polyacrylamide gel (Daiichi Pure Chemicals Co., Ltd., Tokyo, Japan) and subjected to SDS-PAGE. After blotting, PDVF membranes were blocked with a blocking buffer composed of PBS with 5% skim milk and 0.001% Tween. Membranes were then washed 3 times with a washing buffer consisting of PBS plus 0.001% Tween. OPRT and TS primary antibodies were diluted 1:500 (50 ng/mL) and 1:1000 (0.6 μg/mL) in PBS, respectively. After overnight incubation, membranes were washed 3 times with washing buffer and then incubated with anti-rabbit IgG antibody as the secondary antibody included in the ECL Plus Western blotting detection kit (GE Healthcare UK Ltd., Buckinghamshire, England), according to the manufacturer's instructions. After the second antibody reaction, bands were detected using the ECL Plus Western blotting detection kit (GE Healthcare UK Ltd.), according to the manufacturer's instructions.

### mRNA quantification

Real time RT-PCR was performed using a LightCycler™ (Roche Diagnostics K.K., Tokyo, Japan) with a QuantiTect SYBR Green PCR Kit (Qiagen K.K.). Total RNA was isolated from each cell line with ISOGEN (Nippon Gene, Tokyo, Japan), following the manufacturer's directions. Five μg of total RNA was used with ReverTra Ace (Toyobo Co., Osaka, Japan) for reverse transcription. The reaction mixture included 50 ng total RNA. We assayed the expression of OPRT, DPD, TS, thymidine phosphorylase (TP) and UP, the murine homologue of human PyNPase. Primer sequences were as follows: β-actin sense 5'-GCTCTTTTCCAGCCTTCCTT-3' and antisense 5'-TCTCCTTCTGCATCCTGTCA-3'; DPD sense 5'-GACTGAAAGCTGATGGCACA-3' and antisense 5'-TGAATAGCGCTGCATACCTG-3'; OPRT sense 5'-TCCCGAGTAAGCATGAAACC-3' and antisense 5'-TTAGCCGCTGCAAGTATTCC-3'; TS sense 5'-TCTGCTCACAACCAAACGAG-3' and antisense 5'-GCAGAAAATCCCAAGCTGTC-3'; UP sense 5'-TGCTCCAACATCACCATCAT-3' and antisense 5'-ACTGCACCAACTCCTGAACC-3'; and TP sense 5'-TGTTAAGTTTGGGGGAGCTG-3' and antisense 5'-TCTTCCACCTCCAGGGTATG-3'.

Real-time RT-PCR cycles started with 15 min at 95°C and then 50 cycles of 15 sec at 94°C, 20 sec at 56°C and 20 sec at 72°C for β-actin, OPRT, TS and UP. Expression levels of each enzyme were expressed as ratios relative to β-actin expression and compared among cell lines using the L0 parental line as a control.

### Glycoform analysis

Heated and lyophilized cells (1.0 × 10^6 ^cells) were suspended in 0.1 M Tris-HCl buffer (200 μL, pH 8.0) containing 200 μg each of trypsin and chymotrypsin (Sigma-Aldrich Co., St Louis, MO), incubated for 24 h at 37°C, and then heated at 90°C for 10 min to stop enzymatic reactions. N-glycans were released from glycopeptide-containing digests by treatment with N-glycosidase F (20 U, Roche Diagnostics, Tokyo, Japan) for 24 h at 37°C in the same solution. Finally, pronase (200 μg, Calbiochem, Merck, Darmstadt, Germany) was added to the crude mixture and incubated for 24 h at 37°C. Oligosaccharides were purified on a Bio-Gel P-4 column (1.0 × 38 cm, Bio-Rad) with water as the eluant, and sugar-containing fractions were collected and lyophilized. Oligosaccharides were reductively aminated with 2-aminopyridine in the presence of sodium cyanoborohydride (Sigma-Aldrich Co.), according to the method of Hase et al. [47,48]. Pyridylaminated (PA)-oligosaccharides were purified by gel-filtration on a Sephadex G-15 column (Amersham Biosciences, Piscataway, NJ) with 10 mM ammonium bicarbonate as the eluant. Sialic acid residues at the non-reducing termini were selectively released from oligosaccharides by acid hydrolysis at pH 2.0 and 90 °C for 60 min. PA-oligosaccharides were further purified by high performance liquid chromatography (HPLC) (7000 series, Hitachi-High-Technologies Co, Tokyo, Japan) on an amide column (4.6 × 250 mm, TOSOH, Tokyo, Japan). Elution was performed at a flow rate of 1.0 mL per minute at 40°C using two solvent systems composed of 3% acetic acid-triethylamine buffer (pH 7.3) and acetonitrile [A, 35:65 (v/v) and B, 65:35 (v/v)]. The column was initially equilibrated with solvent A, and 7 min after sample injection the eluant was changed to solvent B. Chromatography was monitored by a fluorescence spectrometer (Excitation 320 nm and Emission 400 nm).

Purified PA-oligosaccharides were analyzed by their elution positions on reverse-phase column chromatography (HRC-ODS, 6 × 150 mm, Shimadzu Co, Kyoto, Japan). Each oligosaccharide fraction separated by ODS liquid chromatography was subsequently subjected to analysis on an amide-adsorption column (Amide-80, 4.6 × 250 mm). Products in some peaks were digested with exo-glycosidases and further analyzed using HPLC. As a result, their structures were derived by matching the elution position with the database [34,49].

## Abbreviations

CD-DST- Collagen gel droplet-embedded culture drug sensitivity test;

DPD- Dihydropyrimidine dehydrogenase;

5-FU- 5-fluorouracil;

Fuc- Fucose;

Gal- Galactose;

Glc- Glucose;

GlcNAc- N-acetylglucosamine;

HPLC- High-performance liquid chromatography;

IC-50- 50% inhibition concentration;

Man- Mannose; 

MTT- Mossman's 3-(4,5-Dimethyl-2-thiazolyl)-2,5-diphenyl-2H-tetrazolium bromide;

ODS- Octadecylsilyl;

OPRT- Orotic acid phosphoribosyltransferase;

PA- Pyridylaminated;

PyNPase- Pyrimidine nucleoside phosphorylase;

TP- Thymidine phosphorylase;

TS- Thymidylate synthase;

UP- Uridine phosphorylase.
